# In Vitro Investigation on Degradable Mg-Based Biomaterial under the Impact of the Serum Glycoprotein Fetuin

**DOI:** 10.3390/ma14175005

**Published:** 2021-09-01

**Authors:** Heike Helmholz, Blessing Adejube, Bérengère Luthringer-Feyerabend, Regine Willumeit-Römer

**Affiliations:** 1 Helmholtz Zentrum Hereon, Institute of Metallic Biomaterials, Max Planck St., 21502 Geesthacht, Germany; berenger.luthringer@hereon.de (B.L.-F.); regine.willumeit@hereon.de (R.W.-R.); 2 Faculty of Engineering, Cristian Albrecht University Kiel, Kaiserstraße 2, 24143 Kiel, Germany; blad@tf.uni-kiel.de

**Keywords:** AHSG, biomineralization, implant, magnesium, osteoblast

## Abstract

Biomedical applications of magnesium (Mg) and its alloys are generally dependent on their degradation behavior in vivo. Despite its attractive properties, which make Mg suitable for orthopedic applications, the in vivo material-tissue (bone, blood, and lymph tissues) interaction is not yet fully understood. To investigate the influence of major serum proteins on the degradation, this study focused on fetuin, which is one of the major non-collagenous plasma proteins and which is essential for biomineralization. This study used a physiological setup to investigate the influence of fetuin on the degradation behavior of pure Mg in the presence of calcium (Ca). Extruded pure Mg samples were immersed under cell culture conditions in Hank’s balanced salt solution (HBSS) under defined Ca regimes. The results showed a significant decrease in the degradation rate (DR) when both fetuin and Ca were present in an immersion medium as compared to media where they were not simultaneously present. A possible reason for this behavior was the forming of a dense, protein-degradation products protection barrier at the material surface. Furthermore, the limitation of freely available Ca might be a reason for a decreased degradation. The cultivation of primary osteoblasts (pOB) was possible at the fetuin-coated Mg-surface without additional serum supplementation.

## 1. Introduction

Magnesium (Mg) and its Mg alloys have attracted much research interest due to their lightweight property and their promising characteristics for temporary implant materials [1,2,3]. In addition to the favorable mechanical properties for bone compared to permanent implants of higher stiffness, Mg-based biodegradable implants assist bone healing and stability over a period of time until the implant gradually dissolves, which obviates the need for a second surgery [4,5,6]. Mg and its alloys are highly susceptible to rapid degradation in aqueous environments. This degradation property of Mg could be an advantage or a limitation in biomaterial application depending on the location and expected duration of the implant in the body. Tailoring and controlling the degradation rate by alloying, biochemical-coating, or further surface modification is required for the successful translation into medical application [7]. The in vivo degradation behavior of Mg is yet to be fully understood, and thus a detailed understanding of its in vitro degradation mechanism and interaction with several biological components is necessary to evaluate its in vivo degradation behavior. Sensitive and reliable in vitro biocompatibility tests are a prerequisite for a successful translation into pre-clinical and clinical studies [8]. One reason for the considerable difference of in vitro and in vivo results of Mg degradation might be the interference of serum proteins with the implant [9]. A major serum protein is fetuin-A (α2 Heremans Schmidt glycoprotein AHSG), which is a high-abundant glycoprotein of fetal bovine serum (FBS), a supplement of natural origin used in many in vitro test setups. Fetuin is an acidic serum glycoprotein and a physiological regulator of bone metabolism. It is involved in biomineralization and shows a high affinity for bone minerals [10,11,12]. Presently, there is no information on the interaction of fetuin with Mg-based implants in the presence of bone minerals. Fetuin is a key factor for osseointegration due to its function as Ca-mineral binding protein and a major non-collagenous protein of the extracellular matrix [13,14]. A primary physiological function is the prevention of unwanted soft tissue calcification as well as inhibition of vascular calcification. This protein is a systemic, soluble inhibitor of pathological mineralization and is essential for the correct skeletal formation [15]. Fetuin deficiency has been related to a reduction in vascular-wall elasticity, which is a crucial cardiovascular risk factor in the dialysis population [16,17,18]. The inhibition of mineral precipitation by stabilization of Ca-P and the induction of effective removal of soluble mineral colloid by phagocytic cells from circulation are dominant mechanisms to prevent pathological calcification.

The Mg-based biometals are under discussion as implants for the musculoskeletal system as well as for vascular stents. The interaction with the Ca^2+^ and Ca-P complexing fetuin, which is expressed by mesenchymal stem cells and osteocytes, may influence the biocompatibility and integration of such Mg-based implants. This study aimed to investigate if there is an impact of fetuin on the degradation of Mg in the presence of Ca. Different mechanisms might be possible. Due to the affinity of fetuin for Ca, calciprotein particles (CPP) will be formed [19]. These could then precipitate on the Mg surface, forming a protection layer and/or limiting Ca interaction with the Mg surface, thereby preventing rapid degradation. However, a removal of Ca from the physiological solution by fetuin complexation could increase the degradation [20].

The aim of the investigation was to illustrate the potential interaction of fetuin, bone minerals, and Mg-based biomaterials. The main focus was to show and explain an impact on the degradation behavior in vitro. Consequently, the response of bone forming osteoblasts to the functionalized Mg surface will be shown.

## 2. Materials and Methods

### 2.1. Materials and Chemicals

High purity Mg (99.94 wt.% purity, Luxfer MEL Technologies, Magnesium Electron, Manchester, UK) was molten under a protective atmosphere (argon + 2% sulfur hexafluoride). Permanent mold direct chill casting was used to produce ingots, and, afterwards, indirect extrusion of the ingots at 300 °C was used to produce a 12-mm rod. Discs with dimensions of 10 mm (L) × 10 mm (W) × 1.5 mm (H) were machined from the cast Mg rod. Material casting and processing were performed at the Magnesium Innovation Centre (MagIC), Helmholtz Zentrum Hereon. The chemical composition of the cast material was analyzed in order to identify potential trace contaminations (Table 1). The pure Mg discs were wet-ground using the silicon carbide (2500 grit silicon carbide) abrasive paper at 50 rotations per minute with a twin-wheel grinder and polisher (Saphir 360, ATM Qness GmbH, Mammelzen, Germany). All discs were cleaned ultrasonically for 20 min in n-hexane, 20 min in acetone, and 3 min in 100% ethanol (all chemicals, Merck KGaA, Darmstadt, Germany). Next, samples were sterilized ultrasonically in 70% ethanol for 20 min and finally dried in 24-well cell culture plates under sterile conditions to avoid microbial contamination.

Fetuin from fetal bovine serum was purchased from Sigma-Aldrich Chemie GmbH (Steinheim, Germany). The specific Ca concentration needed was prepared from calcium chloride dihydrate, 99.5% CaCl_2_ × 2H_2_O (Chemsolute TH.Geyer, Rennigen, Germany). For the lectin staining, lectin kit fluorescein (FLK 2100, Vector laboratories Inc., Burlingame, CA, USA) was applied. Nitric acid (HNO_3_) suprapur 65% (Sigma-Aldrich Chemie GmbH, Steinheim, Germany) was used for trace analysis of Ca and Mg, and all dilutions were performed using ultrapure water type I.

### 2.2. Immersion Test

Hank’s balanced salt solution ((HBSS), Sigma-Aldrich Chemie, Steinheim, Germany) modified with sodium bicarbonate, and without phenol red, calcium chloride, and magnesium sulfate, was used as the base solution (control) for all immersion media. The relevant concentrations of fetuin (1, 2, and 3 mg/mL) as well as Ca (2–8 mM) were prepared in HBSS and sterile-filtered (0.02 µm) before the initiation of the immersion test. Media were freshly prepared for each experimental set-up.

The grinded, sterilized Mg discs were immersed in media (1 Mg disc of ~0.16 g in 1 mL) and incubated under cell-culture conditions (37 °C, 5% CO_2_, 20% O_2_, and 95% relative humidity). A semi-static immersion test condition was adopted in this experiment; thus, immersion media were changed every 2 or 3 days until the endpoint. The two endpoints for the immersion time were 7 (run A) and 15 (run B) days. Two different, independent experimental set-ups were performed: (i) constant fetuin but varying Ca concentrations (“run A”) and (ii) constant Ca but two different fetuin concentrations (“run B”). For each run, 9 technical replicates were used. Due to efficiency reasons, supernatants of 6 replicates were used for pH, osmolality, and Mg/Ca determination, and 200 μL of the supernatants were extracted under sterile conditions and stored at −80 °C to be used for protein assay. The discs were separated for surface analysis. A group of three was used for determination of the degradation rate. Another three were applied for surface analysis with electron microscopy (VEGA3 Tescan GmbH Dortmund, Germany and with a Phenom ProX G6 Desktop SEM, Thermo Fisher scientific, Waltham, MA, USA), and the final three were used for lectin staining followed by elemental imaging.

For each media change, pH (pH meter Sentron SI600 pH-meter Fisher Scientific, Schwerte, Germany) and osmolality (Osmometer GonotecTM Osmomat Auto 20 Fisher scientific Berlin, Germany) were determined. Before and after immersion, the weight of each sample was measured (m_1_; m_2_). Three replicates were taken from each condition for determination of the degradation rate (DR). Removal of the degradation products was done by the use of chromic acid (180 g/L chromium (VI) oxide, avantor, Darmstadt, Germany) for 20 min at room temperature followed by cleaning with ultrapure water and 100% ethanol, drying, and, ultimately, the measurement of the final weight (m_2_). The degradation depth (in mm/year) was calculated via the following equation:

DR = (8.76 × 10^4^ (m_1_ − m_2_))/Aρt (1)

where A represents the surface area in cm^2^, ρ is the density of pure Mg (1.74 g/cm^3^), t is the immersion time in hours, m is the observed mass in gram, and DR is the degradation rate in mm/year. It has been shown from previous research that chromic acid treatment will not dissolve bulk pure magnesium, and, so, only the degradation products will be removed [21].

The different conditions can be summarized as follows, and the labelling of the experimental groups was equalized for all diagrams and figures (Table 2).

### 2.3. Surface Analysis

Surface characterization was performed for degraded discs without removal of the degradation products using SEM (VEGA3 Tescan GmbH Dortmund, Germany and with a Phenom ProX G6 Desktop SEM, Thermo Fisher scientific, Waltham, MA, USA). The accelerating voltage and the working distance used to obtain the micrographs were 15 kV and 15 mm, respectively. Energy-dispersive X-ray spectroscopy (EDXS) measurements (100 s scanning duration) were performed using an EDXS detector. To obtain an electroconductive surface for the SEM analysis the bottom edge of each disc was tinted with gold.

The surface-bound fetuin was visualized with wheat germ agglutinin—fluoresceinisothiocyanat conjugate (WGA-FITC). For the lectin staining, the labelling solution was first prepared by dilution of 1.0 mg/mL lectin conjugate stock solution into HBSS. The final concentration was then brought to 10 μg/mL. A volume measuring 70 μL labelling solution was placed on top of the disc to cover the disc surface, after which it was incubated for 30 min at room temperature in the dark. The labelling solution was removed, and the disc washed twice in 1 mL of HBSS. Micrographs were taken by laser scanning confocal microscopy (Leica TSC SP8, Leica Microsystems, Wetzlar, Germany). Large scan images were obtained and merged automatically to cover the whole disc area. Image analysis was done with the LAS X Leica suite software.

Micro-X-ray fluorescence Spectrometry (µXRF) was applied for the elemental imaging of the disc surface. Precipitated elements were visualized by a Tornado M4 (Bruker nano, Berlin, Germany). The disc area was mapped utilizing a rhodium (Rh) tube working at a voltage of 50 kV and an anode current of 600 mA. The fluorescence radiation was detected with a Bruker XFlash^®^ Silicon Drift Detector 30 mm^2^ (Bruker nano, Berlin, Germany), energy resolution < 145 eV for 250.000 cps. The sample chamber was operated under vacuum conditions (20 mbar). The sample surface was mapped with a spot size of 25 nm, a spot distance of 15 mm, and an acquisition time of 1.5 ms per pixel. Image analysis was done with the Bruker ESPRIT microanalysis software version 1.3.0.3273 (Bruker nano, Berlin, Germany).

### 2.4. Supernatant Analysis

A bicinchoninic acid (BCA) protein assay (PierceTM BCA Protein Assay Kit, Thermo scientific, Rockford, IL, USA) for Mg content determination and Ca content determination was used for the characterization of the supernatant.

The standard curve was prepared (using BSA standards provided with the kit) with concentrations ranging from 0.05 mg/mL to 1.00 mg/mL. Standards and samples were prepared in triplicates. The supernatants were diluted with HBSS to the expected concentration range of approximately 0.2 mg/mL. The absorbance was measured in a Tecan microplate reader (Tecan Sunrise TECAN Deutschland GmbH, Crailsheim, Germany) at a wavelength of 562 nm.

Atomic absorption spectroscopy (Agilent 240AA, Waldbronn, Germany) with external 6-point calibration was used for the quantification of Mg^2+^ and Ca^2+^ in the collected portion of each supernatant. Aliquots of the supernatants from 6 (run A) and 4 (run B) technical replicates of each media exchange were used for the measurement and were diluted with 1% HNO_3_ suprapur to ensure a concentration range between 0.05 mg/mL to 1.00 mg/L. For Mg determination, 1% HNO_3_ suprapur was used as a dilution solution, while 1% HNO_3_ 4% lanthanum (La as a releasing agent) was used as dilution solution for Ca determination. Calibration standards were prepared, and the accuracy of the measurement was checked with a single-element reference solution (Roth, Karlsruhe, Germany) and observed to be within 95–105% recovery, which showed a very narrow range of error.

### 2.5. In Vitro Cell Culture Experiments

Primary osteoblasts (pOB) were prepared as described earlier [22,23]. The pOB were adopted to a serum-free culture by using DMEM:F12 (1:1 *v:v*.) supplemented with 1% insulin, transferrin, and selenium supplement solution (ITS) according to the recommendations of van der Valk et al. [24]. After achieving a vital continuous serum-free pOB culture, the cells were pre-differentiated for one week by supplementation of 10 nM dexamethasone, 10 nM dihydroxyvitamine D3, 0.282 nM L-ascorbic acid 2-phosphate, and 5 mM β glycerolphosphat. Mg discs were processed according to the protocol (2.1.) and immersed in HBSS, with 4 mM Ca in HBSS and 2 mg/mL fetuin in 4 mM Ca in HBSS for 48 h in order to obtain a surface coating. The concentrations were chosen according to the results from immersion tests and in order to retain an excess and sufficient amount of fetuin. Uncoated but sterilized glass cover slides were used as control. The pOB were seeded in a density of 40.000 cells/cm^2^, and the medium was changed every 2 to 3 days. The cell viability was tested after 8 days with a 1.6 µM calcein-AM, 2 µM ethidiumbromid homodimer staining. Furthermore, the focal adhesion staining kit (FAK100 merck millipore, Darmstadt, Germany) was used to observe the actin cytoskeleton. Nuclei were visualized by 1 µg/mL Höchst 33342 staining. The OsteoimageTM Mineralization Assay (Lonza group AG, Basel, Switzerland) was applied to document the formation of hydroxyapatite after a cultivation period of 13 days. Microscopic images were taken with the Leica SPM8 confocal microscope and a fluorescence microscope NI eclipse (Nikon, Düsseldorf, Germany).

### 2.6. Statistics

The data used for this study were analyzed and plotted utilizing the Origin 9.0 software (Originlab Corporation, Wellesley Hills, MA, USA). Standard analysis comparing data within the same experiment group was done by using SigmaPlot version 13.0 (Systat software GmbH, Frankfurt a.M, Germany) analysis as well as the Excel statistics add-on Winstat^®^ version 2012.1.0.96 (R. Fitch software, Bad Krozingen, Germany).

## 3. Results

### 3.1. Impact on Degradation

**Figure 1:**
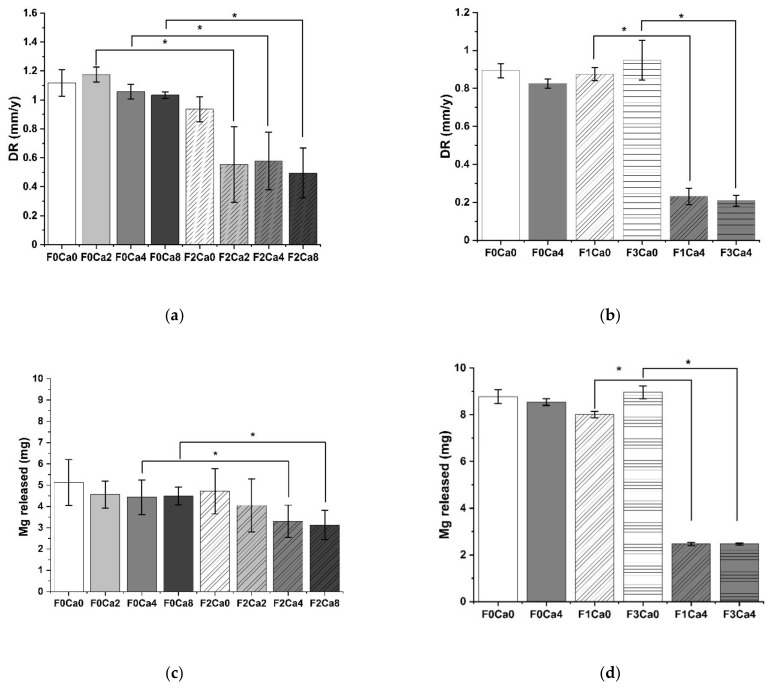
Impact on Mg degradation at final endpoints run A 8 d and run B 15 d; (**a**) degradation by weight loss method run A; (**b**) degradation by weight loss method run B; (**c**) degradation by Mg liberation run A; (**d**) degradation by Mg liberation run B; mean ± SD, * *p* < 0.05, Mann–Whitney U-test; (**a**,**b**) *n* = 3; (**c**,**d**) *n* = 4; F = fetuin concentration in immersion media in mg/mL; Ca = Ca concentration in immersion media in mM.

The determination of the degradation rate by weight loss was applied as an endpoint method to show the overall effect of fetuin on Mg degradation (Figure 1a,b). There was a significant reduction in the DR when both fetuin and Ca were present in the immersion media. Fetuin alone did not induce an inhibitory effect. Increasing concentrations of Ca alone did not show any effect on degradation. Considering these results, a Ca concentration of 4 mM was chosen for further experiments. Surprisingly, it seemed to be an on–off mechanism because concentration above 1 mg/mL fetuin exhibited the same diminishing effect on the Mg DR (Figure 1b). The same observations could be documented by analyzing the absolute amount of lost Mg measured not in the form of mass loss but as released Mg into the immersion medium. For this, elemental analysis was used as a different analytical tool and another kind of approach to evaluate the degradation in physiological conditions (Figure 1c,d). However, the difference was less pronounced utilizing the Mg—mass balance because a significant difference in the absolute amount of released Mg was determined for 4 mM and 8 mM Ca concentrations, proving the necessity of applying alternative analytical tools.

**Figure 2:**
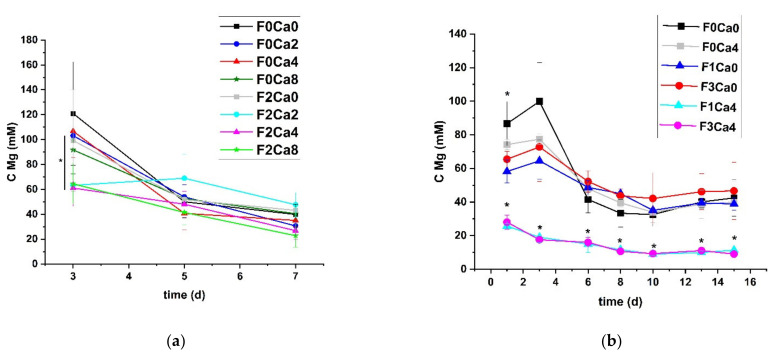
Time-dependent Mg liberation; (**a**) run A; (**b**) run B; mean ± SD, *n* = 4; * *p* < 0.05; Mann–Whitney U-test; (**a**) at day 3 between F0Ca2 and F2Ca2, F0Ca4 and F2Ca4, and F0Ca8 and F2Ca8; (**b**) day 1 between F0Ca0 and F0Ca4, day 1–15 F1Ca0 and F1Ca4, and F3Ca0 and F3Ca4. F = fetuin concentration in immersion media in mg/mL; Ca = Ca concentration in immersion media in mM.

In order to follow the kinetics of the degradation process, the liberation of Mg into the immersion media was determined. In the first experiment, the smoother Mg release with fetuin in combination with Ca became obvious (Figure 2a). There was an almost constant level of 30 mM to 70 mM Mg at each medium exchange when applying both Ca and fetuin. Since there was already observed a significant difference between samples immersed with and without fetuin at day 3, a more detailed investigation was performed starting with Mg determination after 24 h of immersion. The inhibition of the Mg release by the fetuin-Ca combination could be verified even after the first day (Figure 2b). Furthermore, it was confirmed that there was no dependency on fetuin concentration. After a first phase of fast Mg release until day 6, a continuous degradation within the only Ca-containing-or the only fetuin-containing controls was detected. By contrast, a slight but continuous Mg liberation with only a marginally higher Mg concentration at day 1 was observed for samples containing both Ca and fetuin factors. The differences between treatment groups with and without fetuin were significant over the whole experiment duration. Additionally, there was a slight but significant decrease in Mg release at day 1 comparing the immersion in pure HBSS and HBSS with 4 mM Ca.

**Figure 3:**
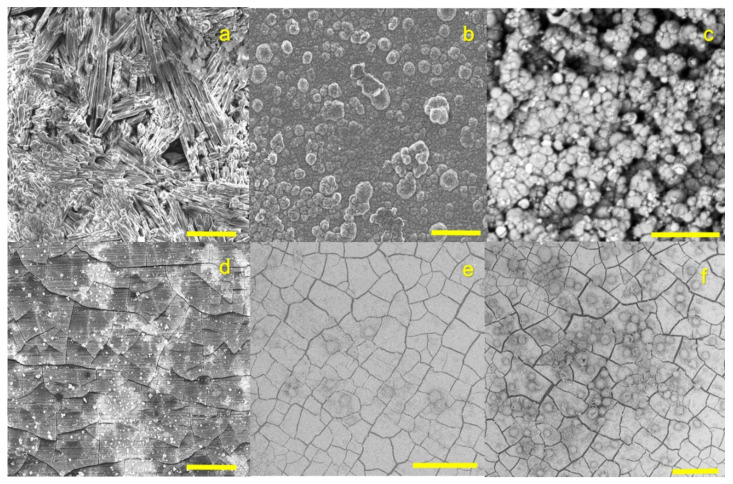
Surface morphology after immersion in different degradation media under physiological conditions. (**a**) HBSS 15 d; (**b**) 3 mg/mL fetuin in HBSS 15 d; (**c**) 4 mM Ca in HBSS 15 d; (**d**) 3 mg/mL fetuin in 4 mM Ca in HBSS 15 d; (**e**) and (**f**) surfaces prepared with 2 mg/mL fetuin in 4 mM Ca in HBSS with pOB after 7 d of serum-free cell culture; two different positions scale bar: (**a**,**b**) and (**d**–**f**) 100 µm; (**b**) 200 µm.

According to the variations in degradation behavior, the degradation–surface morphology appeared to be quite different, and the impact of the various solutions on Mg degradation could be visualized by SEM (Figure 3). A compact layer could only be detected when both Ca and fetuin were present. When HBSS was applied as a control, rod-like crystals were observed. Immersion separately in fetuin resulted in loosely packed layers reflecting protein aggregates. This was verified by exemplary EDXS analysis (Table 3) where the degradation products generally comprised Mg, oxygen (O), carbon (C), phosphorus (P), and sodium (Na). As expected, Ca was present only in media prepared with 4 mM Ca, which was confirmed by the µXRF elemental analysis. The high amount of Mg and O in HBSS indicated that the corrosion product primarily consisted of MgO (and also Mg(OH)_2_). Detection of C could indicate the formation of magnesium carbonate (MgCO_3_).

### 3.2. Distribution of Ca

**Figure 4:**
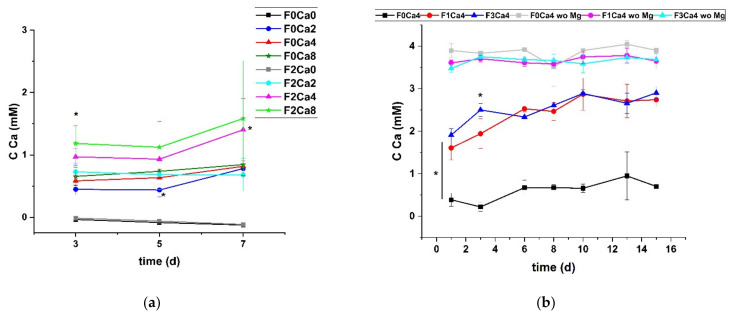
Time-dependent Ca concentration; (**a**) run A; (**b**) run B; mean ± SD, *n* = 4; F = fetuin concentration in immersion media in mg/mL; Ca = Ca concentration in immersion media in mM wo Mg = media control without Mg surface; * *p* < 0.05 Mann–Whitney U-test; (**a**) at day 3 between F0Ca2 and F2Ca2, F0Ca4 and F2Ca4, and F0Ca8 and F2Ca8; at day 5 between F0Ca2 and F2Ca2; (**b**) day 1 between F0Ca0 and F0Ca4, day 1–15 F1Ca0 and F1Ca4, and F3Ca0 and F3Ca4.

**Figure 5:**
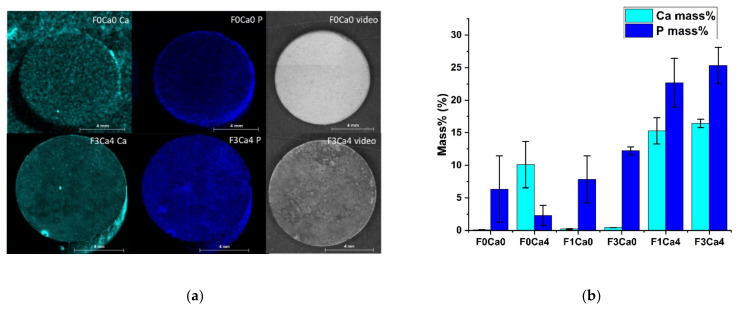
Elemental analysis of Ca and P deposition at the surface after 15 d in immersion medium utilizing µXRF; (**a**) cyan Ca, blue P, and the video image of two exemplary extreme conditions: pure HBSS (F0Ca0) and fetuin 3 mg/mL in 4 mM Ca in HBSS (F3Ca4); (**b**) element quantification at disc surface in mass% mean ± SD, *n* = 2.

In order to characterize the fate of supplemented Ca within the degradation process in dependency of the Ca-binding protein fetuin, the Ca content was determined in the supernatant as well as on the material surface. Figure 4 shows the continuous removal of free Ca^2+^ independent of the supplemented Ca concentration in the presence of an Mg surface. The addition of 2 mg/mL fetuin induced an increase in Ca in the immersion medium, which indicated a capture function of this protein (Figure 4a). This effect was significantly pronounced at day 3 for all Ca concentrations (2, 4, and 8 mM), but it continued until day 7 for a Ca concentration of 8 mM. The data in Figure 4a verified the absence of Ca in the controls. In order to prove the fetuin-dependent effect, the second run was performed for 15 days. Three groups of treatments can be clearly distinguished from Figure 4b. At first, the concentration of the supplemented Ca remained constant at 4 mM without any adsorbing Mg surfaces (without = wo Mg) as expected. The majority of the supplemented Ca was continuously removed from the supernatant without fetuin (F0Ca4), indicating a permanent binding at the degrading Mg surface. Furthermore, a significantly elevated Ca concentration was determined in the immersion media under the presence of 1 and 3 mg/mL fetuin. Although Ca and fetuin were supplemented continuously with each media exchange, a saturation effect could not be observed because the Ca did not reach the 4 mM basis concentration. A Ca trapping effect was hypothesized, which was independent of the fetuin concentration up to 3 mg/mL. The related deposition of Ca at the Mg surface was analyzed by an end-point method utilizing micro-X-ray fluorescence spectrometry. The element imaging is exemplarily presented in Figure 5a, with the two extremes HBSS (F0Ca0) and fetuin 3 mg/mL in 4 mM Ca in HBSS (F3Ca4). The quantification of run B (varying fetuin concentration) is summarized in Figure 5b. The intensity for Ca at the HBSS (F0Ca0)-treated surface was in the range of the background value, which was undetectable. The Ca deposition was increased with addition of fetuin. This effect seemed to be independent of the applied fetuin concentration, as shown in Figure 5b (F1Ca4; F3Ca4). Since the mineralization process required the deposition of Ca in conjunction with P, this element was also quantified. A clear positive correlation between Ca and P sedimentation at the Mg surface was found. It seemed that only fetuin already bound a higher portion of P compared to the pure material control in the absence of Ca. Interestingly, the bound Ca fraction without fetuin supplementation was increased compared to the adjacent P. The data support the hypothesis of the occurrence of two potential effects: an increased Ca sedimentation but also a stabilization of Ca content in the immersion medium.

### 3.3. Distribution of Fetuin

**Figure 6:**
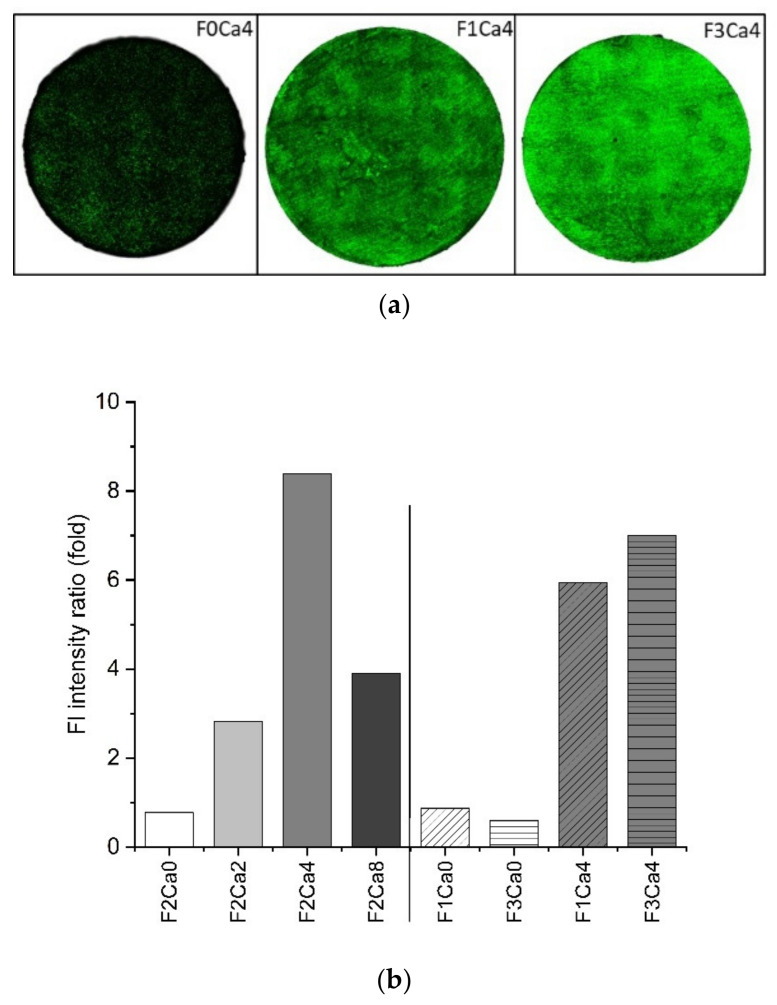
Fetuin analysis by FITC-conjugated WGA lectin staining at the surface after 8 and 15 d in immersion medium; (**a**) microscopic images of three exemplary conditions: background F0Ca4, F1Ca4 1 mg/mL fetuin in 4 mM Ca in HBSS, and F3Ca4 fetuin 3 mg/mL in 4 mM Ca in HBSS; (**b**) quantification of fluorescence intensity averaged over disc area, expressed as ratio between blank sample without fetuin and the corresponding sample with 2 mg/mL (run A left part *n* = 3) and 1 and 3 mg/mL fetuin (run B right part *n* = 2) as fold increase.

Quantifying the adsorption and distribution of fetuin is another key parameter to describe the impact of this glycoprotein on Mg degradation. Therefore, a carbohydrate side-chain-specific lectin staining was performed to document the adsorption of fetuin on the material surface. The fluorescence intensity in the microscopic images in Figure 6a represents the amount of bound fetuin. A quantitative evaluation was performed by calculating the ratio of fluorescence intensity indicating the increase of adsorbed fetuin with increasing concentrations of Ca (Figure 6b). There was no quantitative binding of fetuin at the pure Mg surface detectable without the addition of Ca. The fetuin concentration in the supernatant was measured utilizing the BCA protein assay in order to verify the microscopic results on the material surface. Comparing the dependency of fetuin concentration on Ca supplementation (Table 4), no significant effect could be observed. Since there were no time dependent variations, the fetuin concentration was averaged over the study duration. There was a slightly higher concentration in the 8 mM Ca supernatant, which reflected the lower adsorption observed at the surface. The portion of adsorbed fetuin was, in general low, comparing the concentrations in the supernatants with and without the Mg disc. The recovery rate for fetuin was about 83% without and 70% with Mg, respectively. The Ca supplementation at the lower fetuin concentration of 1 mg/mL induced a significant increase in this protein in the medium supernatant.

### 3.4. Interaction with Primary Osteoblasts

**Figure 7:**
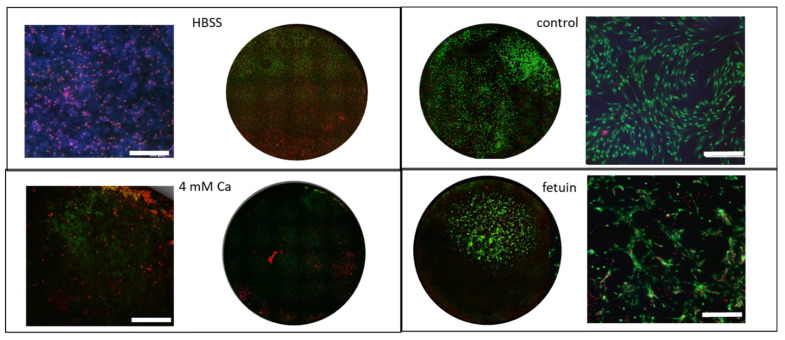
Live/dead staining after 8 d of serum-free in vitro culture of primary osteoblasts on Mg samples treated 4 mM Ca in HBSS and 2 mg/mL fetuin in 4 mM Ca in HBSS; glass surface as control; green—calcein AM (vital); red—ethidiumbromid homodimer (dead); blue—Hoechst 33342 (nucleus); overview disc area and close up scale bar: 500 µm.

**Figure 8:**
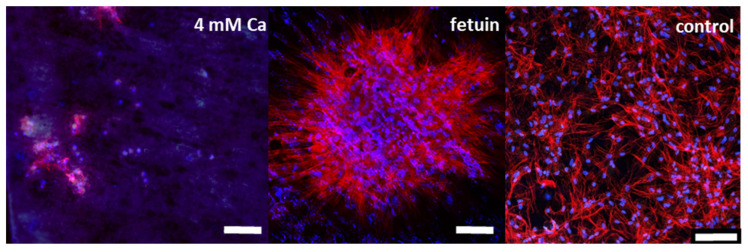
Actin (red)/nucleus (blue) staining after 8 d of serum-free in vitro culture of primary osteoblasts on Mg samples treated with 4 mM Ca in HBSS and 2 mg/mL fetuin in 4 mM Ca in HBSS; glass surface as control; scale bar: 100 µm.

**Figure 9:**
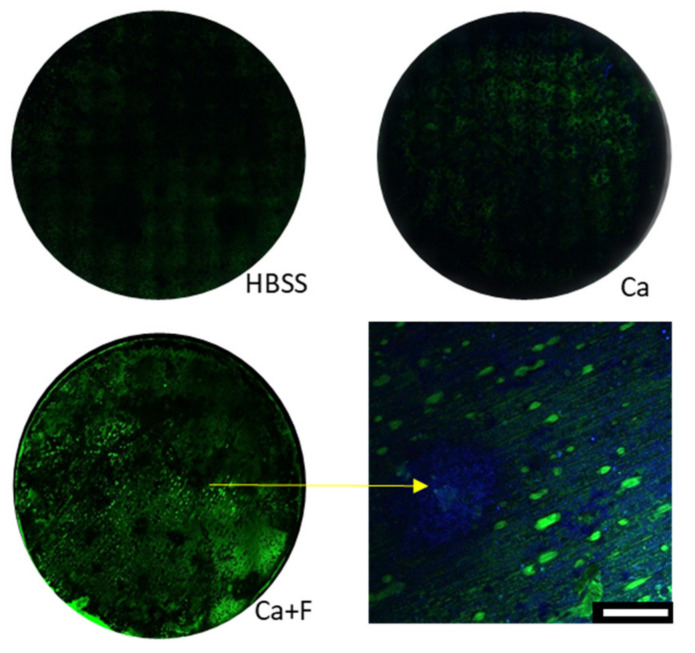
HA (green) staining by Osteoimage© after 13 d of serum-free in vitro culture of primary osteoblasts on Mg samples treated with HBSS, 4 mM Ca in HBSS, and 2 mg/mL fetuin in 4 mM Ca in HBSS; micrographs of disc area and close-up of the 2 mg/mL fetuin in 4 mM Ca sample; blue—Hoechst 33342 staining of cell nuclei; scale bar: 250 µm.

Fetuin is a high-abundant protein in fetal calf serum. In order to avoid interferences, it was essential to develop a serum-free pOB culture to ensure defined experimental conditions. This was achieved with a 1:1 (*v:v*) mixture of DMEM:F12 supplemented with ITS. The adopted pOB were seeded on the pre-treated Mg discs with a viability of >85%. The cells were used in a pre-differentiated state. The live/dead staining clearly indicated no vital cells at the Mg discs with HBSS (Figure 7). The pOB at the surface of fetuin-treated samples that aggregated to cell nodules showed a good viability compared to the very low number of vital cells at the Ca 4 mM Mg surface. However, compared to the control surface, the number of viable cells was lower, and the cells were sparsely distributed. A staining of the actin cytoskeleton was applied in order to show the adhesion and morphology of the pOB on Mg in a serum-free culture (Figure 8). The little remaining pOB at the 4 mM Ca surface appeared rounded and small. By contrast, the pOB on the fetuin surface had an elongated inner structure reflecting the surface shape of the material. As already observed with the viability staining, the pOB aggregated to local colonies. The mineralization capability measured after two weeks of cultivation by staining of hydroxyapatite did not show a remarkable production of the pOB attached at the fetuin surface (Figure 9). The maintenance of the pOB at the Mg surface under the serum-free culture conditions seemed to influence the metabolic capability. Therefore, it is necessary to optimize the serum-free pOB culture to enable a temporal extension and observe a clear mineralization over time.

The amount of released Mg was used as an indicator for the impact of material pre-treatment and cell growth on material degradation.

A release of 6.36 mg (±0.25 mg) of absolute Mg was measured for HBSS-treated Mg discs after 13 days of cell culture. A remarkably lower Mg liberation of 3.93 mg (±0.75 mg) and 2.02 (±0.05 mg) was determined for the 4 mM Ca and the corresponding 2 mg/mL fetuin coating, respectively. These results confirmed the degradation inhibition of the fetuin treatment. Especially in this treatment group, there was a significant difference comparing the Mg release of Mg-based discs with and without cells (Table 5). While there was a constant liberation with pOB at a lower level, the release of Mg into the cell culture medium was already elevated in the control group without pOB at day 2, and it increased over time.

## 4. Discussion

The degradation rate of an Mg bone-implant material may to some extent depend on its interaction with collagenous and non-collagenous serum proteins. Studying the unique behavior of major serum proteins at the implant surface as a factor for improved osseointegration could aid in bridging the gap between in vivo and in vitro degradation results. The focus of this study was to investigate the influence of fetuin on the degradation of pure Mg in the presence of Ca. Fetuin was selected as a high-abundant protein in fetal bovine serum and because of its major regulatory function for biomineralization.

HBSS, which was the base medium used in this study, has been assessed to be a defined medium applied for in vitro immersion tests [25]. It was utilized as a base in order to simulate physiological concentration. Its ionic content has also been evaluated to be similar to that of the blood plasma with a high chloride concentration [26]. This medium was also selected to ensure a defined Ca concentration to be set in the physiological level (<2 mM), but it reflected a hypercalcemic status as well [27].

There was no significant impact on the degradation rate within this concentration range, which is in contrast to previous findings [20]. However, in combination with fetuin there was a significant reduction in the Mg degradation measured either by weight loss or by Mg release.

The complex process of Mg degradation in vitro is extensively discussed and described [28,29]. According to the composition of HBSS, degradation products like Mg(OH)_2_ and MgCO_3_ are expected to accumulate on the Mg surface, limiting the mass transfer of ions between the Mg substrate and the solution and thereby acting as a barrier against degradation [30]. With the presence of Ca, the formation of loosely packed Ca-P precipitates was possible, as shown with the elemental analysis and electron microscopy.

After implantation, biomaterials are immediately faced to a multitude of proteins. They are key factors to determine the later success or failure of an implant. The general behavior of proteins and small organic molecules was reviewed by Höhn et al. (2019) [31]. However, fetuin was not specifically considered in this summary. In general, protein adsorption on Mg surfaces is a multifactorial process where chemical composition of the surface, surface roughness, hydrophilicity and wettability, as well as the composition of the corrosion media play an important role. The lectin-staining of the adsorbed fetuin demonstrated a Ca–concentration dependency with a saturation above a Ca concentration of 4 mM. Since the surface was polished and cleaned in the same manner, no material-related effects were observed.

The investigation of protein adsorption and its impact on Mg degradation were performed with albumin, the most-abundant serum protein, or fetal bovine serum itself as a protein mixture. Hou et al. (2019) showed a gradual degradation inhibition of BSA compared to the whole serum. Furthermore, the inhibitory effect was more pronounced with the addition of Ca [32]. These results are in agreement with the present investigation where a remarkable attenuation effect of fetuin was only observed in combination with Ca. When present in an alkaline solution, fetuin is negatively charged, and when the medium solution contains PO_4_^3−^, the negatively charged protein will compete for adsorption to the positively charged Mg surface. Loosely bound precipitates could be seen at the Mg surface by treatment with fetuin that still permitted the ion transfer and degradation processes to occur. A tight and compact degradation barrier could only be observed when both Ca and fetuin were present simultaneously in the immersion media. Due to the high affinity of fetuin for Ca–P complexes, CPP were formed, which are relatively stable. However, the in vitro experiments showed that the degradation can be induced again when transferring the material into serum-free cell culture conditions. Proteins can have multiple effects on metal corrosion. Besides the barrier effect reducing the degradation, there is also the potential complexation of metal cations reducing the concentration of free ions and therefore accelerating the corrosion [33]. Although both processes may occur depending on the environmental conditions, the barrier effect prevailed in the present study.

The concentrations of fetuin used in this study were higher compared to normal physiological conditions, but they represented the concentration range when 10% FBS was used in in vitro studies when the immersion media contained serum [34]. Furthermore, these concentrations were selected in order to obtain clarity on its effects on the degradation of an implant material and to gain an understanding of the local concentration effect of fetuin on an implant in vitro. Previous studies revealed that, in the presence of other acidic serum proteins, fetuin concentrations of <0.1 mg/mL can stabilize CPP. Further studies on this discovered that patients with a fetuin concentration below 0.2 mg/mL faced a high mortality risk because low fetuin-A serum concentration was tightly associated with calcification and cardiovascular mortality in several patients [10,35]. Dialysis patients suffer from fetuin-A deficiency, which may be a reason why in comparison to the general population these patients face a greater risk of cardiovascular calcification-associated morbidity and mortality. In the present study, an elevated Ca concentration was determined in the immersion medium only under the impact of fetuin and in the presence of Mg as an adsorption surface. However, the supplied immersion medium concentration of 4 mM was not achieved, indicating an ongoing adsorption at the Mg surface. Without fetuin, the Ca concentration in the immersion medium was lower at an almost stable level of 0.5 mM, demonstrating the development of a Ca-containing adsorption/degradation layer on the Mg surface. With fetuin, more Ca was retained in the supernatant. This result is in accordance with previous reports that fetuin binds Ca and transports these as CPP in the blood, thereby preventing calcification of soft tissue [16,36]. The higher Ca content in the presence of fetuin supported the hypothesis of a trapping of Ca by fetuin in the medium.

The results are based on a multitude of different analytical methods focusing on both the immersion supernatants and material surface processes. This lowers the simultaneous processing of a higher number of technical replicates, which would be preferred to achieve a better statistical significance.

Protein adsorption on medical devices is a key process for the consequent recruitment and adhesion of cells. The cell–material interaction depends on the mediation by proteins. The adhesion of pOB was one issue of the present investigation due to the promising application of Mg-based implants in bone-repair processes.

Most in vitro experiments demonstrating the biocompatibility of Mg-based degradable materials ensure a cell adsorption via a pre-incubation of the material in serum containing cell culture media [37]. However, under such conditions it is not possible to evaluate the impact of selected serum components. Therefore, a serum-free pOB culture was generated, which provided a continuous, acceptable viability of more than 85%. A serum-free osteoblast progenitor cell model for biomaterial testing was introduced by Duewelhenke and Eysel (2007) [38]. It has been shown that fetuin, likewise to the well-known adhesion proteins fibronectin and vitronectin, can function as an adhesion promoter on titanium materials. Serum albumin had an opposite effect. In this study, Ultroser SF was used as a FBS replacement, but it may also contain animal products [39]. In the present study, only defined but basal nutrient solutions with supplementation of insulin and transferrin were applied, according to the recommendations of the working group for FBS replacement [24]. Under these conditions, the detection of viable cells on the fetuin surface was possible; however, there was no homogenous distribution of the cells on the surface, and aggregates were formed. The adhesion might be mediated by annexins on the cell surface [40]. The cells were able to adhere and spread, as shown by the actin cytoskeleton staining. Although the fetuin surface was acceptable for the pOB, there was a huge difference to the well-compatible glass surface. Optimization of seeding and cell culture media conditions might be necessary to improve the viability of the pOB and the repeatability of the serum-free, direct-contact assay.

Besides the adhesion, the function of the pOB could also be influenced by fetuin. It has been shown that naturally synthesized fetuin is a factor for osteogenic differentiation of mesenchymal stem cells in conjunction with the fibroblast growth factor FGF23. Fetuin was expressed in pre-mature OB, not released from mature OB, and increased again during further differentiation to osteocytes [41,42]. Binkert et al. (1999) suggested a gradient regulatory function of fetuin on osteogenesis by binding TGF-β and consequently inhibiting mineralization in dexamethasone activated bone marrow cells [43]. Although no increased activity of alkaline phosphatase (data not shown) as well as no remarkable synthesis of hydroxyapatite were detected in the present study, detailed investigation is necessary to confirm the relation of artificially provided fetuin to osteoprogenitor cells, their proliferation, and their function under the impact of Mg-based biomaterials.

Future investigations should include a wide variety of serum- and glycoproteins in defined mixtures to correlate molecular characteristics to the impact on material degradation. The present study targeted a major and high-abundant serum protein with a proven effect on biomineralization, and an impact on Mg degradation was shown for the first time. However, serum is a much more complex mixture of multi-functional proteins acting as chelating or adhesive molecules. The consequent adhesion of osteoblasts responsible for bone remodeling needs more detailed investigation because the serum-free cell-culture model has to be optimized and described in detail in order to assess the effect of functionalized Mg on cell adhesion and mineralization processes.

## 5. Conclusions

This study contributes to the research towards achieving a bio-functional, clinically beneficial Mg implant for therapeutic clinical application. The biological environment, especially the specific impact of various serum proteins is one of the key factors for understanding the degradation of Mg and might be helpful to clarify degradation processes in vitro and in vivo. In the case of fetuin, a potential Ca-P functionalization of the Mg surface or the application of Mg-Ca alloys might be advantageous to enrich the surface with this serum glycoprotein, which consequently serves as a linker for an improved cell adhesion. By contrast, the demonstrated Ca-complexation effect of fetuin might be a potential advantage to prevent unwanted vascular calcification within the scope of therapeutical application as stents in cardiovascular diseases.

## Figures and Tables

**Table 1:** Chemical composition of pure Mg samples determined by optical emission spectrometry.

Composition (Wt.%)	Ca	Zn	Fe	Cu	Si	Al	Mn
Weight percentage	0.0041	0.0018	0.0048	0.0003	0.016	0.016	0.020

**Table 2:** Summary of experimental conditions.

Sample	C Fetuin (mg/mL)	C Ca (mM)	Run
F0Ca0	-	0	A/B
F0Ca2	-	2	A
F0Ca4	-	4	A/B
F0Ca8	-	8	A
F2Ca0	2	0	A
F2Ca2	2	2	A
F2Ca4	2	4	A
F2Ca8	2	8	A
F1Ca0	1	0	B
F3Ca0	1	0	B
F1Ca4	3	4	B
F3Ca4	3	4	B

**Table 3:** Exemplary EDXS analysis (atomic %) obtained from the disc areas after 15 d of immersion (n.d.—not detected).

Sample	C (%)	O (%)	Mg (%)	P (%)	Ca (%)
F0Ca0	22.35	55.10	20.26	2.37	n.d.
F3Ca0	22.81	53.52	23.30	n.d.	n.d.
F3Ca4	29.45	45.04	6.55	7.55	7.57

**Table 4:** Fetuin analysis by BCA protein assay in immersion medium averaged over time—7 d (run A) and 15 d (run B); mean ± SD; *n* = 4; * *p* < 0.05, Mann–Whitney U-test; <LOD below limit of determination.

Sample	Cav Fetuin (mg/mL)	SD
F2Ca0 8 d	1.22	0.14
F2Ca2 8 d	1.26	0.08
F2Ca4 8 d	1.27	0.23
F2Ca8 8 d	1.41	0.16
F0Ca0 15 d	<LOD	
F0Ca4 15 d	<LOD	
F1Ca0 15 d	0.39	0.10
F1Ca4 15 d	0.73 *	0.17
F3Ca0 15 d	2.03	0.19
F3Ca4 15 d	2.14	0.20

**Table 5:** Time-dependent Mg liberation during serum-free in vitro culture of primary osteoblasts on Mg samples treated with 2 mg/mL fetuin in 4 mM Ca in HBSS; mean (±SD), *n* = 3; * *p* < 0.1; Mann–Whitney U-Test.

Time (days)	With pOB C Mg (mM)	Without pOB C Mg (mM)
2	21.18 (3.86) *	32.10 (1.92)
6	21.82 (1.90) *	57.34 (1.09)
8	17.11 (0.61) *	43.32 (11.18)
13	23.01 (1.98) *	50.42 (12.21)

## Data Availability

The data presented in this study are available on request from the corresponding author. The data are not publicly available due to ongoing research and planned commercialization.
